# What Is behind Changes in Resting Heart Rate and Heart Rate Variability? A Large-Scale Analysis of Longitudinal Measurements Acquired in Free-Living

**DOI:** 10.3390/s21237932

**Published:** 2021-11-27

**Authors:** Marco Altini, Daniel Plews

**Affiliations:** 1 Department of Human Movement Sciences, Vrije Universiteit Amsterdam, De Boelelaan 1105, 1081 HV Amsterdam, The Netherlands; 2 Sports Performance Research Institute New Zealand (SPRINZ), AUT University, 17 Antares Place, Rosedale, Auckland 0632, New Zealand; daniel.plews@aut.ac.nz

**Keywords:** heart rate variability, heart rate, training, stress, sickness, menstrual cycle

## Abstract

The aim of this study was to investigate the relationship between heart rate and heart rate variability (HRV) with respect to individual characteristics and acute stressors. In particular, the relationship between heart rate, HRV, age, sex, body mass index (BMI), and physical activity level was analyzed cross-sectionally in a large sample of 28,175 individuals. Additionally, the change in heart rate and HRV in response to common acute stressors such as training of different intensities, alcohol intake, the menstrual cycle, and sickness was analyzed longitudinally. Acute stressors were analyzed over a period of 5 years for a total of 9 million measurements (320±374 measurements per person). HRV at the population level reduced with age (*p* < 0.05, r = −0.35, effect size = moderate) and was weakly associated with physical activity level (*p* < 0.05, r = 0.21, effect size = small) and not associated with sex (*p* = 0.35, d = 0.02, effect size = negligible). Heart rate was moderately associated with physical activity level (*p* < 0.05, r = 0.30, effect size = moderate) and sex (*p* < 0.05, d = 0.63, effect size = moderate) but not with age (*p* = 0.35, r = −0.01). Similar relationships between BMI, resting heart rate (*p* < 0.05, r = 0.19, effect size = small), and HRV (*p* < 0.05, r = −0.10, effect size = small) are shown. In response to acute stressors, we report a 4.6% change in HRV (*p* < 0.05, d = 0.36, effect size = small) and a 1.3% change in heart rate (*p* < 0.05, d = 0.38, effect size = small) in response to training, a 6% increase in heart rate (*p* < 0.05, d = 0.97, effect size = large) and a 12% reduction in HRV (*p* < 0.05, d = 0.55, effect size = moderate) after high alcohol intake, a 1.6% change in heart rate (*p* < 0.05, d = 1.41, effect size = large) and a 3.2% change in HRV (*p* < 0.05, d = 0.80, effect size = large) between the follicular and luteal phases of the menstrual cycle, and a 6% increase in heart rate (*p* < 0.05, d = 0.97, effect size = large) and 10% reduction in HRV (*p* < 0.05, d = 0.47, effect size = moderate) during sickness. Acute stressors analysis revealed how HRV is a more sensitive but not specific marker of stress. In conclusion, a short resting heart rate and HRV measurement upon waking using a smartphone app can effectively be used in free-living to quantify individual stress responses across a large range of individuals and stressors.

## 1. Introduction

Autonomic control impacts heart rhythm in response to stress [1,2,3,4]. In particular, the heart has its own pacemaker, beating at approximately 100 beats per minute. However, heart rate at rest is typically lower than the intrinsic firing rate of the sinoatrial node (the pacemaker) due to the influence of the autonomic nervous system (ANS) via its two main branches: the parasympathetic system and the sympathetic system. Normally, the parasympathetic branch of the ANS slows down heart rate and increases heart rate variability (HRV), while the sympathetic branch of the ANS increases heart rate and reduces HRV [1,5]. When measuring physiology in a resting state, there are differences between resting heart rate and HRV due to increased parasympathetic influence during different phases of the cardiac cycle [6]. For example, parasympathetic activity is higher during the exhale phase of the breathing cycle [7,8]. Additionally, the influence of the parasympathetic system on heart rhythm is almost instantaneous [3,9], resulting in large differences in beat-to-beat heart rate. However, by definition, resting heart rate averages out any beat-to-beat differences in consecutive heartbeats. As a result, parasympathetic modulation in response to stress tends to be better captured by HRV with respect to heart rate [10]. For these reasons, resting heart rhythm and, in particular, HRV, have been widely investigated in relation to acute stressors.

When considering training as an acute stressor, the rationale behind monitoring recovery using resting heart rate or HRV is therefore coupled with how the ANS responds to such stressors. In the context of physical exercise, intense training shifts the ANS towards a sympathetic dominance [11,12,13], which is reflected in the higher heart rate and in the lower HRV 24–48 h after training [11,14]. Reductions in HRV and increases in heart rate as measured at rest first thing in the morning on the day after high-intensity aerobic exercise have been reported across a wide range of individuals [10,14]. However, heart rate increases after training are often very small and of limited practical applicability [10,15]. Similarly, other stressors have been investigated acutely. For example, alcohol intake was reported to suppress HRV while impacting heart rate to a lesser extent [16]. On the other hand, resting heart rate has been proposed as a clear marker able to detect infections as well as recovery from an infection [17], changes that typically are also reflected in HRV [18]. When analyzing acute stressors, it is of interest to establish whether the relationship between resting heart rate, HRV, and the stressor is reproducible across a wide range of individuals. Unfortunately, most studies to date focused on a homogeneous sample, typically of male and relatively young individuals [19], therefore limiting our understanding of the relationship between, e.g., training, sickness, alcohol intake, and resting heart rate and HRV in other groups of the population. In the context of a longitudinal analysis in response to stressors, the menstrual cycle should also be considered, given that several studies have shown how HRV is slightly suppressed during the luteal phase of the menstrual cycle [20].

Apart from the mechanisms influencing heart rhythm in response to stress, stratifying population-level data across different subgroups of individuals (e.g., based on sex, activity level, age, or body mass index (BMI)) can provide useful insights into the differences between resting heart rate and HRV. For example, previous research has highlighted how HRV reduces with age [21]. Additionally, the link between cardiorespiratory fitness and resting heart rate seems stronger than for HRV, despite a few studies showing increased HRV in response to an exercise program [22]. Typically, women have higher resting heart rates than men [23]. It follows from the inverse relationship between resting heart rate and HRV that HRV should be slightly lower in women. However, according to published literature [24], this is not necessarily the case.

In recent times, monitoring resting heart rate and HRV unobtrusively in real-life settings, outside of the lab, has finally become a practical possibility. Data can be acquired using validated smartphone apps [25,26] longitudinally over periods of weeks or months, providing novel insights on an individual’s response to training and lifestyle stressors. As a result, monitoring physiological stress and recovery status by means of an HRV measurement is becoming more common among elite athletes as well as sports enthusiasts [15]. However, while technological advancements have made it very easy to acquire high-quality heart rate and HRV data in resting conditions, many questions remain unanswered when it comes to the use of resting heart rate and HRV as well as their differences both at the population level and within individuals (i.e., in response to stressors).

Thus, the aim of this cross-sectional and longitudinal analysis is to provide a more comprehensive view of the relationship between resting heart rate, HRV, acute stressors, and population-level characteristics, analyzing data acquired on a large sample of individuals in real-life settings.

## 2. Materials and Methods

### 2.1. Data Acquisition

Physiological data (resting heart rate and HRV) and annotations (individual characteristics and stressors) were collected using the HRV4Training app, as detailed below.

#### 2.1.1. Heart Rate and HRV

**Figure 1:**
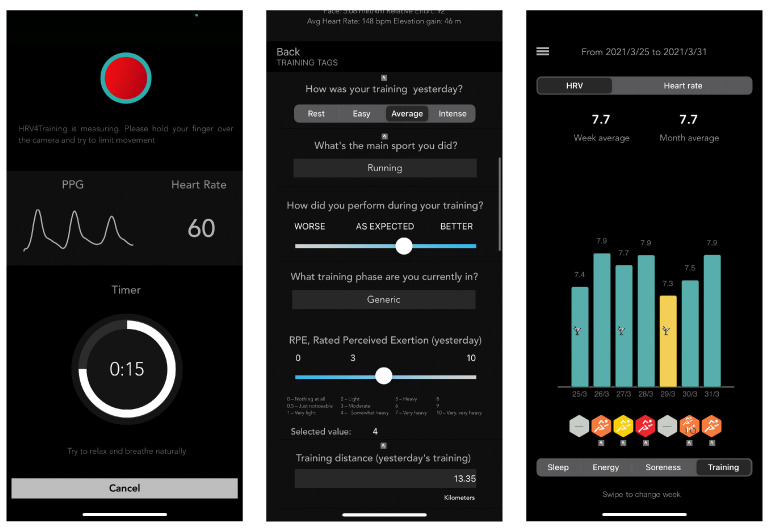
Screenshots of the HRV4Training app. The image on the left side shows the measurement screen, displaying the photoplethysmographic signal acquired via the mobile phone camera. The middle image shows an example of the questionnaire that is used after the measurement to annotate stressors such as training intensity, alcohol intake, sickness, or the menstrual cycle. The third image, on the right, shows a historical view of the data.

Resting heart rate and HRV were collected daily using the validated HRV4Training app [25,26,27]. The HRV4Training app is a commercially available mobile phone app that allows for non-invasive measurements of resting heart rate and HRV using either the phone camera or an external sensor (see Figure 1). Users included in this study downloaded the HRV4Training app from the Apple Store or Google Play out of their own interest and explicitly agreed to provide collected measurements and annotations for research purposes via a consent form embedded in the app. The app instructed users to perform the measurement right after waking up while still lying down to limit the effect of other stressors (e.g., caffeine intake or physical activity). Instructions were provided to reproduce conditions similar to measurements at rest in supervised settings. HRV features that are representative of parasympathetic activity and that are typically reported in the scientific literature are the high-frequency power (HF) and the square root of the mean squared difference between beat-to-beat intervals or rMSSD [28]. However, rMSSD might be preferable as it is less dependent on breathing rate [29] and better standardized. Thus, only rMSSD is reported in this work. Measurement duration was configurable between 1 and 5 min since 1 min measurements were previously validated and considered of sufficient duration for accurate HRV analysis of time-domain features such as rMSSD [30,31].

#### 2.1.2. Users

**Figure 2:**
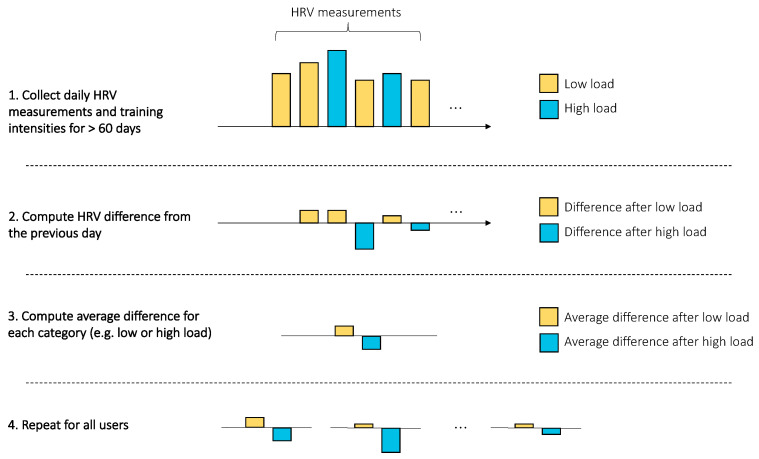
Procedure used for the analysis of acute stressors. First, HRV (and resting heart rate) data and annotated training intensities were collected. Then, day-to-day differences in HRV (and resting heart rate) were computed. Differences were then averaged across categories, e.g., to compute the average day-to-day change in HRV (or heart rate) in response to either easy or high training intensity. The procedure is repeated for each individual so that we can determine the stress response for each stressor at the population level.

Users with at least 60 resting heart rate and HRV measurements were included in the analysis so that individual responses to various acute stressors (alcohol intake, sickness, the menstrual cycle, and training) could be investigated in relation to multiple instances of the same stressors, as opposed to individual instances typically reported in laboratory studies (see Figure 2). Additionally, all measurements that resulted in more than 10% RR intervals being discarded after applying the artefact correction method described in [25] were excluded. After applying the inclusion criteria, 28,175 users (22,750 male and 5425 female) were included, for a total of 9,032,749 measurements (320 ± 364 measurements per user). Mean age across the entire population was 37 ± 12 years, and mean BMI was 23.8 ± 3.2 kg/m${}^{2}$. The user-reported training habits were as follows: 667 users reported training occasionally, 1551 users reported training 1–2 times per week, 11,973 users reported training 3–4 times per week, and 13,614 users reported training daily. The BMI categories were underweight (BMI below 18.5 kg/m${}^{2}$, *n* = 314), normal (BMI between 18.5 kg/m${}^{2}$ and 25 kg/m${}^{2}$, *n* = 16,838), overweight (BMI between 25 kg/m${}^{2}$ and 30 kg/m${}^{2}$, *n* = 6808), and obese (BMI above 30 kg/m${}^{2}$, *n* = 1145).

#### 2.1.3. Individual Characteristics

Users filled in a questionnaire upon registering an account in the HRV4Training app, including individual characteristics analyzed in this paper. In particular, users reported their age, physical activity level (one of the following: not training, training occasionally, training 2–3 times per week, training 4–5 times per week, and training daily), sex, weight, and height, from which BMI was derived.

#### 2.1.4. Acute Stressors

Training days were manually annotated in the app while answering a short questionnaire, which is shown to the user right after the measurement. Training intensities were selected among four categories: rest, easy, average, and intense. Training intensities were then clustered in two groups: low intensity, comprising rest days and training days annotated as easy, and high intensity, comprising training days annotated as average or intense by users (see Figure 2). While training can be quantified in many different ways, the main goal of the proposed clustering was to quantify the effect of low intensity against high-intensity exercise on resting physiology, as typically adopted in a polarized training model [32]. Additionally, users reported alcohol intake, menstruation days, and sickness.

### 2.2. Data Analysis

#### 2.2.1. Population Level Analysis

Individuals were clustered into different subgroups, two for sex (male/female), four for age (20 to 30 years old, 30 to 40 years old, 40 to 50 years old, and 50 to 60 years old), and four for fitness level (training occasionally, 1–2 times per week, 3–4 times per week, or daily). Linear models using either heart rate or HRV as dependent variables and individual characteristics (sex, physical activity level, age, and BMI) as independent variables were built to determine the variance explained by such models.

#### 2.2.2. Analysis of Acute Stressors

**Figure 3:**
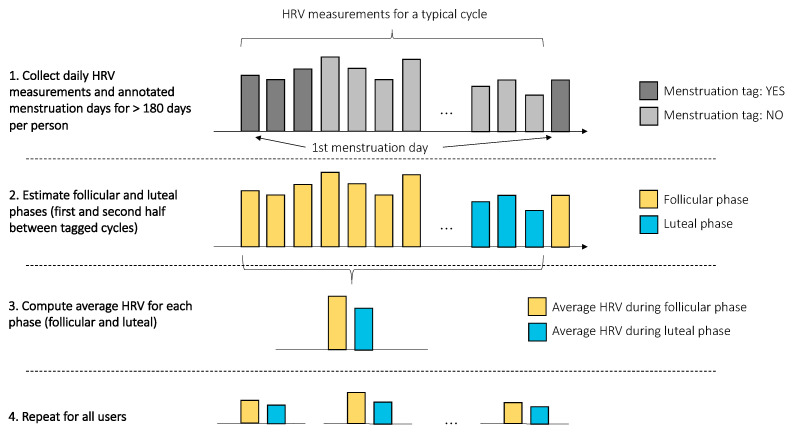
The procedure used for the analysis of the menstrual cycle. First, HRV (and resting heart rate) data, and annotated menstruation days were collected. Then, the beginning of each cycle was defined as the first menstruation day, and the following days, up to the next cycle, were split into two to estimate the follicular and luteal phases. Average heart rate and HRV were computed for each phase (follicular and luteal) and for each user.

The relation between physiological data and training was analyzed by first computing day-to-day differences in heart rate and HRV for each individual. Subsequently, the change in resting heart rate and HRV on days following training of different intensities was analyzed for each user (see Figure 2). Additionally, the relationship between resting heart rate, HRV, and training was analyzed by age group and sex. The same procedure was used to analyze the relationship between heart rate, HRV, and alcohol intake. For sickness data, the percentage change was computed with respect to the non-sick condition. For the menstrual cycle, user-reported menstruation days were used to define the duration of a cycle and to determine the change in resting heart rate and HRV during the follicular and luteal phases, with respect to the users’ average (see Figure 3).

#### 2.2.3. Statistics

Summary statistics are reported as mean ± standard deviations for each measure and subgroup. Mean resting heart rate and HRV were computed for each individual. The results for all acute stressor analyses are reported in percentage with respect to the user’s average heart rate and HRV. Reporting the results as percentages can ease interpretation and comparison of the sensitivity of each marker with respect to a specific stressor. Comparisons between two groups for the population level analysis as well as for acute stressors were carried out using t-tests, with a significance level of 0.05. One-way ANOVA was used to compare three or more groups. Effect sizes are reported using Cohen’s d (d) or Pearson’s correlation coefficient (r) [33,34].

## 3. Results

### 3.1. Population Level Analysis

**Figure 4:**
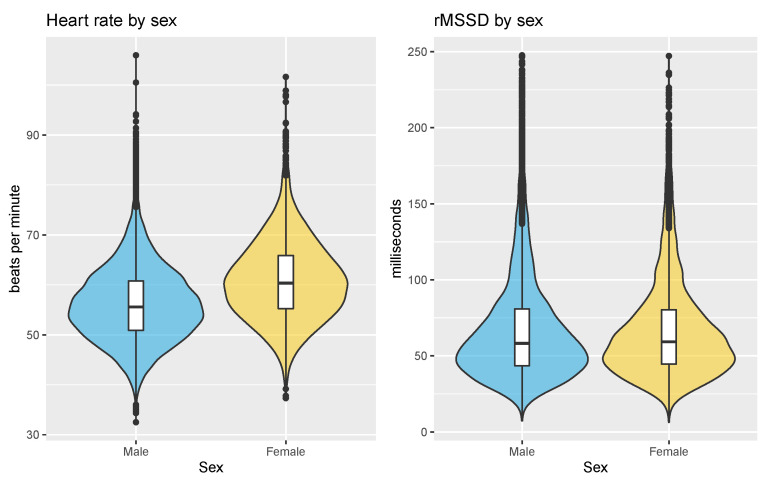
Resting heart rate and HRV by sex. Heart rate is on average 5 bpm higher in female users, while HRV is very similar between male and female users, on average.

The mean rMSSD over the entire dataset was 69 ± 37 ms while the mean heart rate was 57 ± 8 bpm. The resting heart rate and HRV clustered by sex are shown in Figure 4. Heart rate was 56 ± 7 bpm for male users and 61 ± 8 bpm for female users (*p* < 0.05, d = 0.63, effect size = moderate). rMSSD was 67 ± 34 ms for male users and 68 ± 34 ms for female users (*p* = 0.35, d = 0.02, effect size = negligible).

**Figure 5:**
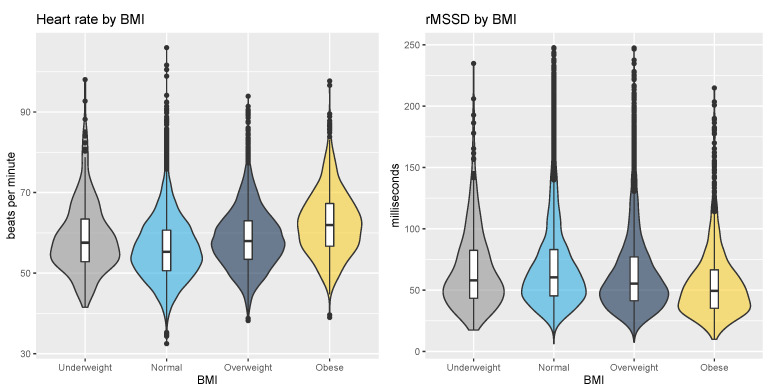
Resting heart rate and HRV by BMI. Resting heart rate is the lowest for the normal category, and similarly, HRV is the highest for the normal category. The largest deviation for both heart rate and HRV is found in the obese category, with the highest resting heart rate and lowest HRV.

Resting heart rate and HRV clustered by BMI are shown in Figure 5. Heart rate was 59 ± 9 bpm for the underweight BMI category, 56 ± 8 bpm for the normal BMI category, 59 ± 7 bpm for the overweight BMI category, and 62 ± 8 bpm for the obese BMI category (*p* < 0.05, r = 0.19, effect size = small). rMSSD was 67 ± 34 ms for the underweight BMI category, 69 ± 35 ms for the normal BMI category, 64 ± 34 ms for the overweight BMI category, and 56 ± 30 ms for the obese BMI category (*p* < 0.05, r = −0.10, effect size = small).

**Figure 6:**
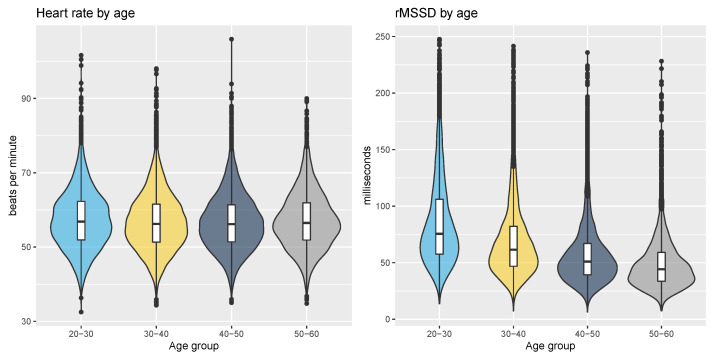
Relationship between heart rate, HRV and age by age group. Resting heart rate does not change across age groups while HRV is clearly reduced.

There was almost no correlation between heart rate and age (*p* = 0.35, r = −0.01), while the correlation between rMSSD and age was moderate (*p* < 0.05, r = −0.35, effect size = moderate). The relationships between heart rate, rMSSD, and the four age groups used in this study are shown in Figure 6.

**Figure 7:**
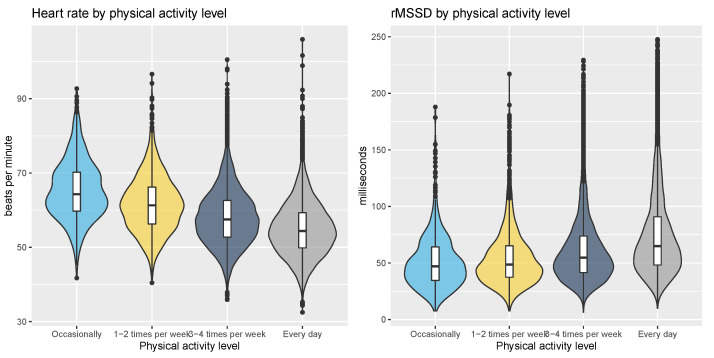
Relationship between heart rate, HRV, and physical activity level. While both resting heart rate and HRV show more positive physiological profiles for the most active individuals, the relationship is stronger for resting heart rate.

The correlation between heart rate and physical activity level was computed as the square root of the explained variance of a linear model, where the dependent variable was heart rate and the independent variable was physical activity level, resulting in r = 0.30 (*p* < 0.05, effect size = moderate). For rMSSD, the correlation with physical activity level was r = 0.21 (*p* < 0.05, effect size = small). The relationship between heart rate, rMSSD, and physical activity level is shown in Figure 7.

**Figure 8:**
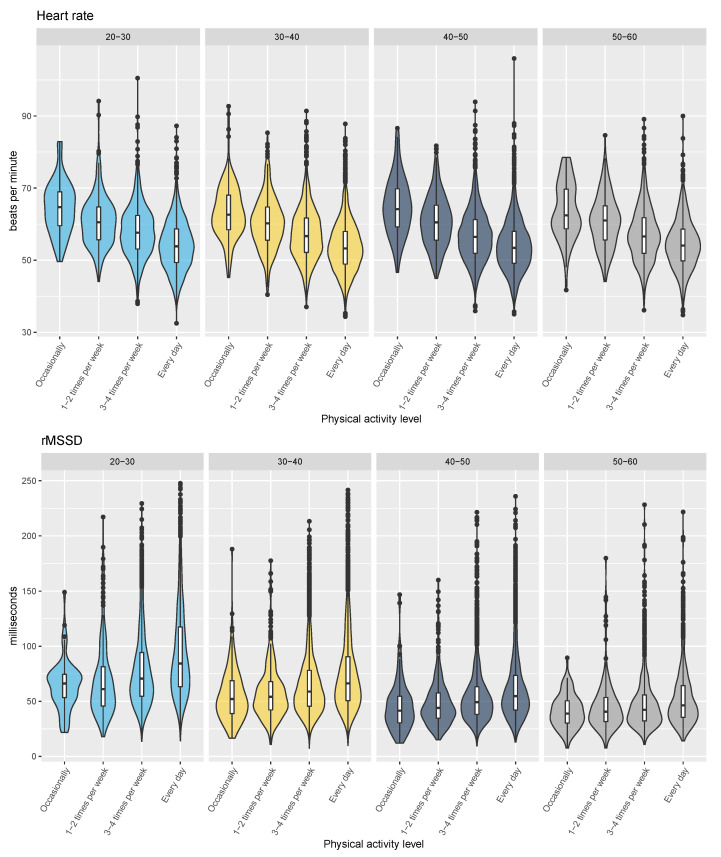
Relationship between resting heart rate, rMSSD, and both age and physical activity level. The association between heart rate and physical activity level remains strong across all age groups, while for rMSSD, it becomes weak for older individuals.

In Figure 8, the relationship between resting heart rate, rMSSD, and both age and physical activity level is shown. The correlation between resting heart rate and physical activity level remains in the range between 0.29 and 0.31 for each age group, while the correlation between rMSSD and physical activity level is the strongest for the youngest age group (r = 0.22 for the 20- to 30-year-olds) but decreases to r = 0.13 for the 50 to 60 years old group. Finally, population-level variables (sex, physical activity level, age, and BMI) can explain 19% of the variance in resting heart rate and 15% of the variance in rMSSD.

### 3.2. Analysis of Acute Stressors

**Figure 9:**
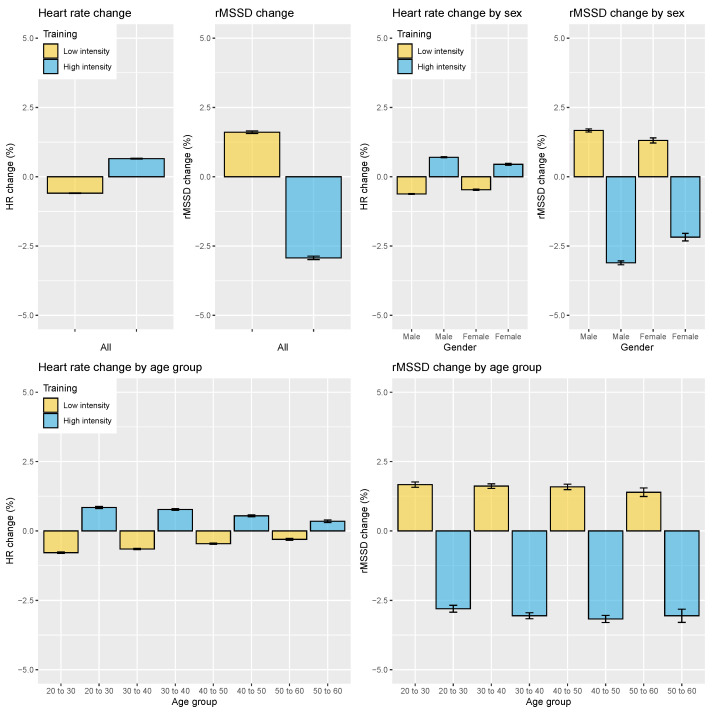
Relation between HR, HRV, and training load split into two categories, analyzed on the entire dataset and grouped by sex or age group. HR is consistently increased on days following higher intensity training load, while rMSSD is consistently reduced. Relative changes in rMSSD are larger, highlighting how HRV can be more discriminating for training intensity. Additionally, percentage changes in heart rate reduced with age while remaining constant for rMSSD. Error bars indicate the standard error.

Acute responses to training were evaluated for two cases—low vs. high training intensity—as well as for the four-category split—rest, low, average, or high training intensity. For the low vs. high intensity split, overall, across all age groups and participants, the heart rate change between low- and high-intensity sessions was 1.3% (*p* < 0.05, d = 0.38, effect size = small). In particular, heart rate was reduced by 0.6% after low-intensity training but was increased by 0.7% after high-intensity training. On the other hand, the rMSSD change between low-intensity and high-intensity sessions was 4.6% (*p* < 0.05, d = 0.36, effect size = small). In particular, rMSSD was reduced by 3% after high-intensity training and was increased by 1.6% after low-intensity training. The results as well as sex and age differences are shown in Figure 9. Female responses were slightly, smaller while the change in heart rate further reduced with age, going from 0.8% for the 20–30 age group to 0.3% for the 50–60 age group (*p* < 0.05). rMSSD reductions after high-intensity training remained fairly constant across age groups (2.8–3.2%, *p* = 0.54).

**Figure 10:**
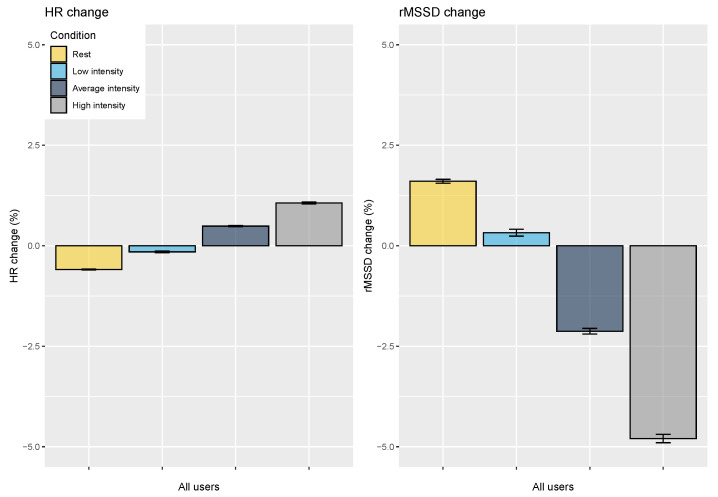
Relation between HR, HRV, and training intensity split into four categories. HR is consistently increased on days following higher training intensity, while rMSSD is consistently reduced. Relative changes in rMSSD are larger, highlighting how HRV can be more discriminative of training intensity. Error bars indicate the standard error.

The results for the four-category split are reported in Figure 10, showing an improved physiological profile after rest days (−0.6% heart rate, +1.6% rMSSD, *p* < 0.05) and increased physiological stress after hard training (+1% heart rate, −4.8% rMSSD, *p* < 0.05).

**Figure 11:**
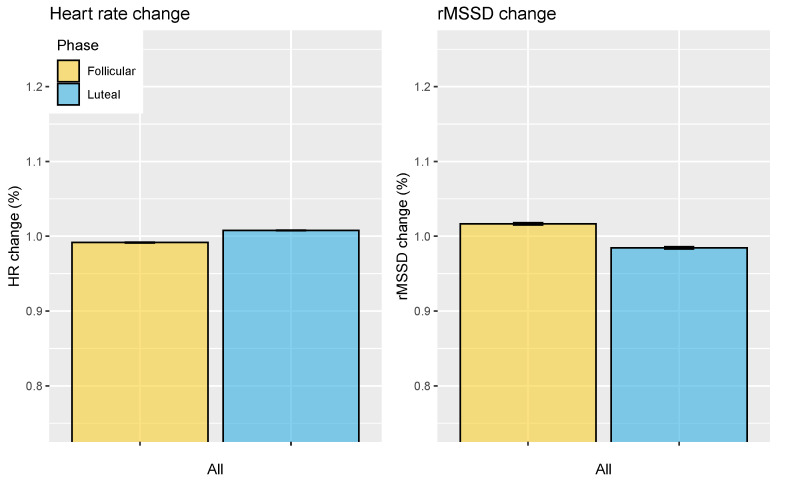
Relation between HR, HRV, and the menstrual cycle. In particular, the difference between the follicular and luteal phases, with respect to an user’s average heart rate and HRV, is reported.

For the menstrual cycle analysis, users that annotated at least five cycles were included, which resulted in 639 users. The results are shown in Figure 11, where changes across all cycles are reported for all included users. Resting heart rate differed by 1.6% between the follicular and luteal phases, with an increase across the cycle (*p* < 0.05, d = 1.41, effect size = large). On the other hand, rMSSD changed by 3.2% between the follicular and the luteal phase, with a decrease across the cycle (*p* < 0.05, d = 0.80, effect size = large).

**Figure 12:**
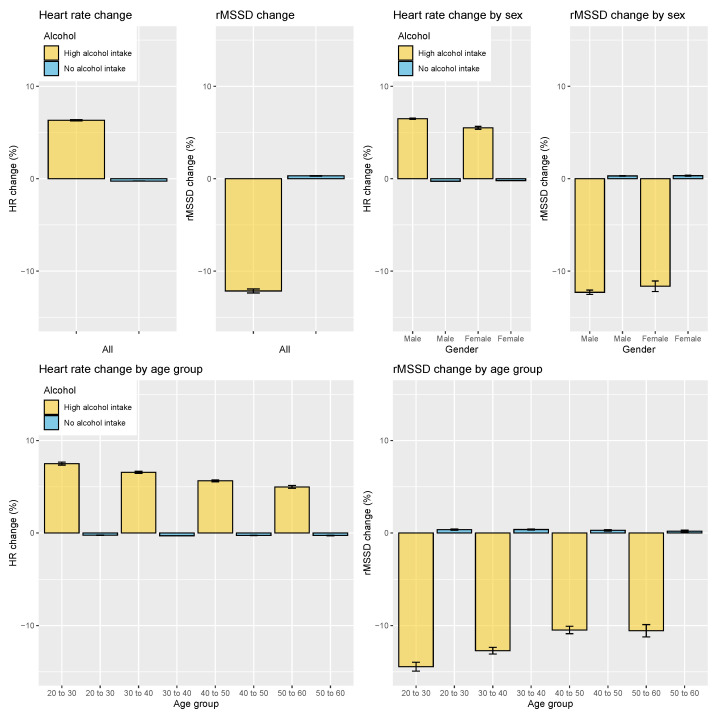
Relation between HR, HRV, and alcohol intake on the entire dataset and grouped by sex or age group. HR is consistently increased on days following higher alcohol intake, while rMSSD is consistently decreased. Error bars indicate the standard error.

In terms of responses to alcohol intake, overall, across all age groups and participants, heart rate increased by 6% after high alcohol intake (*p* < 0.05, d = 0.97, effect size = large), while rMSSD reduced by 12% after high alcohol intake (*p* < 0.05, d = 0.55, effect size = moderate; see Figure 12). A similar response was found in male and female users, while a decrease in the sensitivity to the stressor was shown with increasing age.

**Figure 13:**
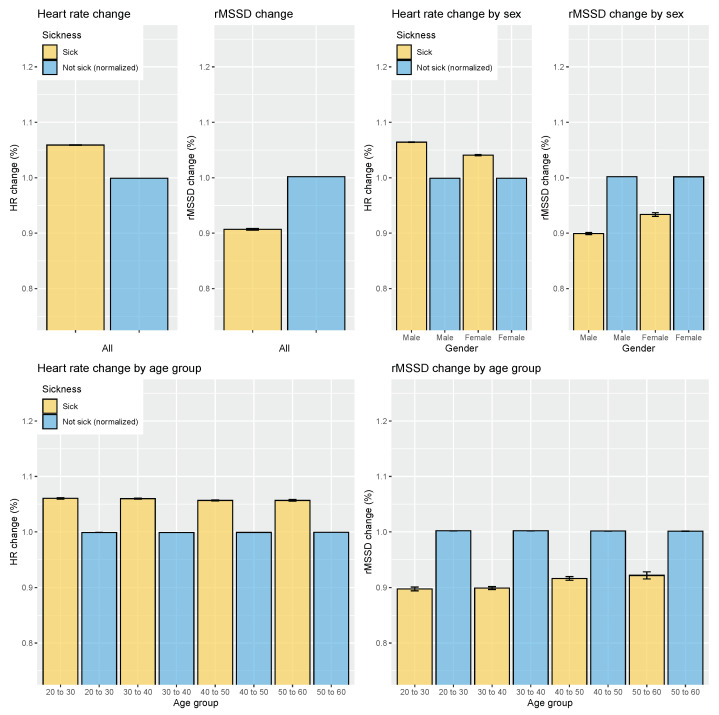
Relation between HR, HRV, and sickness intake on the entire dataset and grouped by sex or age group. HR is consistently increased when sick, while rMSSD is consistently decreased when sick. Additionally, percentage changes in heart rate remain similar across age groups, while they reduce for rMSSD. Error bars indicate the standard error.

For sickness, overall, across all age groups and participants, heart rate increased by 6% with respect to when not sick (*p* < 0.05, d = 0.97, effect size = large), while rMSSD reduced by 10% when sick with respect to the baseline category of not sick (*p* < 0.05, d = 0.47, effect size = moderate; see Figure 13).

## 4. Discussion

In this paper, the relationship between heart rate and HRV with respect to individual characteristics as well as acute stressors, was analyzed in a large sample of 28,175 individuals in free-living. Data acquisition and analysis were standardized by collecting measurements upon waking and by analyzing resting heart rate and rMSSD as the only HRV features. The relationship between heart rate, HRV, age, sex, BMI, and physical activity level was analyzed cross-sectionally, resulting in novel insights such as the reduced association between HRV and physical activity level with older age, which does not impact heart rate. Additionally, the change in heart rate and HRV in response to common acute stressors such as training of different intensities, alcohol intake, the menstrual cycle, and sickness was analyzed over a period of up to 5 years per person. Acute stressors analysis revealed how HRV is a more sensitive but not specific marker of stress. Below, our findings are discussed.

### 4.1. Population Level Analysis

The analysis of the relationship between resting heart rate, HRV, and population-level characteristics such as sex, age, BMI, and physical activity level highlighted important differences between measurements of resting physiology. In particular, at the population level, HRV reduces with age (*p* < 0.05, r = −0.35, effect size = moderate) and is weakly associated with physical activity level (*p* < 0.05, r = 0.21, effect size = small) and not associated with sex (*p* = 0.35, d = 0.02, effect size = negligible), while heart rate shows a stronger association with physical activity level (*p* < 0.05, r = 0.30, effect size = moderate) and sex (*p* < 0.05, d = 0.63, effect size = moderate) but shows no relationship with age (*p* = 0.35, r = −0.01). These findings confirm what was previously reported in laboratory studies and shed additional light on the relationship between resting physiology and population-level characteristics.

It is well known that age is the strongest parameter behind differences in HRV at the population level, while it has a lower impact on heart rate [35]. A low HRV is seen in aging individuals as well in chronic disease and might be associated with autonomic dysfunction or with the deterioration of underlying regulatory mechanisms [35]. Novel insights into the relationship between resting physiology and physical activity level are reported in this work. In particular, at a younger age, a stronger association between HRV and physical activity level was present (r = 0.22). However, this relationship is reduced for older age groups (r = 0.13), as shown in Figure 9. The reason behind the diminishing relationship between physical activity level and HRV with age is unknown. However, a plausible explanation may be due to the relationship between hypervolemia with exercise and baroreceptor sensitivity. It is well established that a potent stimulant for hypervolemia is endurance exercise. Indeed, 24–48 hours post-exercise, there have been observed increases in blood plasma volume (BPV) [36]. Previous literature showed a large correlation between the relative change in rMSSD and change in blood plasma volume (r = 0.85) during recovery from exercise [37]. Such relationships between BPV and HRV are observed due to the increased blood volume and mean arterial pressure resulting in decreased sympathetic outflow to the sinoatrial node and increase in parasympathetic activity [38]. As individuals age, baroreceptor sensitivity is reduced [39], likely reducing the association between increases in BPV and increases in HRV. This may result in the diminished relationship between HRV and physical activity level observed in this study with aging. On the other hand, our data show that the relationship between resting heart rate and physical activity level remains relatively strong and consistent for each age group (r = 0.29–0.31). The relationship between physical activity and resting heart rate is well established, with lower resting heart rates typically reported in response to a training program. Physiologically, as the heart becomes larger, stroke volume increases and cardiac output can be the same at a lower heart rate. This process does not necessarily impact HRV [40]. Furthermore, as resting heart rate is less affected by transient changes in BPV, the relationship between increases in physical activity level and lower resting heart rate is plausibly maintained as populations age.

A similar association between BMI, resting heart rate (*p* < 0.05, r = 0.19, effect size = small) and HRV (*p* < 0.05, r = −0.10, effect size = small) was reported, with a suboptimal physiological profile (higher heart rate and lower HRV) associated with both overweight and underweight categories. When analyzing the relationship between age, resting heart rate, and HRV in relation to sex, similar results were reported for male and female users. These results are expedcted and consistent with published literature.

Finally, linear models with either resting heart rate or HRV as dependent variables and all population level characteristics—age, sex, BMI, and physical activity level—as independent variables were fitted. For both heart rate and HRV, the variance explained by age, sex, BMI, and physical activity level was relatively low (19% for heart rate and 15% for rMSSD). These results are expected given the known influence of genetic factors on heart rhythm [41,42]. Additionally, the lower explained variance for HRV is consistent with previous research highlighting how genetics can explain up to 60% of the variability in HRV metrics, while environmental and health parameters typically explain less of this variance (e.g., 11% in [43]). This is consistent with our experience and current analysis, where even large differences in physical activity level had a minor impact on HRV. On the other hand, HRV was able to capture day-to-day stressors within individuals with high sensitivity, and might therefore be better suited for such day-to-day stress assessment, as covered in the next section.

### 4.2. Acute Stressors Analysis

The relationship between resting heart rate, HRV, and acute stressors such as training, sickness, and alcohol intake was analyzed. The sensitivity of resting heart rate and HRV towards the detection of such stressors is reported, including breakdowns by sex and age. It is important to note that, when looking at acute stressors, oversimplifications are introduced. Any given day, multiple stressors will play a role (exercise, alcohol intake, sickness, as well as other stressors that might not have been tracked, e.g., travel, psychological stressors, etc.), and therefore, it is not possible to isolate acute day-to-day responses to a single stressor outside of controlled laboratory environments. However, this issue was mitigated by including at least two months of data per person, with an average of almost one year of daily measurement per person, so that the response to the same stressors could be analyzed repeatedly over time. The effectiveness of this approach is shown in the results reported, as the physiological responses to isolated stressors are consistent with those in published literature. In particular, an analysis of acute stressors revealed how HRV is a more sensitive marker of stress with respect to heart rate, as shown by larger relative changes. Our data showed a 4.6% change in HRV (*p* < 0.05, d = 0.36, effect size = small) and a 1.3% change in heart rate (*p* < 0.05, d = 0.38, effect size = small) in response to training, a 6% increase in heart rate (*p* < 0.05, d = 0.97, effect size = large) and a 12% reduction in HRV after high alcohol intake (*p* < 0.05, d = 0.55, effect size = moderate), a 1.6% change in heart rate (*p* < 0.05, d = 1.41, effect size = large) and a 3.2% change in HRV (*p* < 0.05, d = 0.80, effect size = large) between the follicular and luteal phases of the menstrual cycle, and a 6% increase in heart rate (*p* < 0.05, d = 0.97, effect size = large) and 10% reduction in HRV (*p* < 0.05, d = 0.47, effect size = moderate) during sickness. Despite the higher sensitivity of HRV, effect sizes were typically larger for resting heart rate, potentially due to how HRV is impacted more in response to stressors not included in this work (i.e., due to lack of specificity).

#### 4.2.1. Strength of the Stressor

When it comes to the strength of the stressor, clear differences are reported. High alcohol intake and sickness trump changes due to training or the menstrual cycle, as these are much greater stressors that typically occur on less frequent occasions. This is somewhat expected as physiology should typically reflect homeostatic control and, therefore, be relatively stable over time. When it comes to training, an improved physiological profile after rest days (−0.6% heart rate, +1.6% rMSSD) and increased physiological stress after hard training (+1% heart rate, −4.8% rMSSD) was reported. Percentage changes in rMSSD are approximately 3–4 times larger than changes in resting heart rate across the various categories, while effect sizes are rather similar. Given that HRV is a more sensitive marker (both physiologically and, as derived in the current dataset, showing a larger percentage change for all stressors), day-to-day changes might be due to a number of stressors. The similar effect size for resting heart rate and HRV responses in the context of training (and reduced effect size for HRV when it comes to other stressors) could be due to the lack of specificity of HRV with respect to a given stressor. On the other hand, heart rate changes are minor and typically only associated to strong stressors, therefore resulting in larger effect sizes. Finally, when clustering the training response by age group, the change in heart rate reduces with age. This reduction seems to point out how assessing training responses and guiding training based on heart rate alone could be ineffective for older individuals. On the other hand, the change in HRV remains constant for each age group, and HRV could therefore be more helpful in guiding training at any age. Breakdown by sex of the reported acute responses shows minor differences, with slightly smaller changes for women in response to all stressors analyzed. The reason behind this difference could be due to changes in resting heart rate and HRV associated with the menstrual cycle. Changes across the menstrual cycle were analyzed in this work using annotated menstruation days. Both heart rate and HRV were impacted, resulting in a less favorable physiological profile during the luteal phase of the menstrual cycle, consistently with published literature [20]. These changes might explain part of the loss of sensitivity in resting heart rate and HRV in response to other stressors. However, the difference could also simply be due to other characteristics of the sample analyzed due to the nature of this observational study.

#### 4.2.2. Interpretability

When considering any index that is used to indicate a need for an intervention or changes in behavior, both the signal (how much the index changes) and noise (the reliability and error), are important considerations. For example, whilst an index may have a low typical error of estimate, if the index has limited change with a given stressor, it is difficult to draw meaningful conclusions and therefore influence behavioral change. The typical errors of an estimate for resting heart rate and HRV (rMSSD) are 10% and 12%, respectively [28]. The signal-to-noise ratio is very close at 0.7 and 0.8, with a slightly better signal-to-noise ratio for HRV, while the reported effect size in this study is also similar between resting heart rate and HRV. Such data are very important, as they allow practitioners to establish thresholds to identify meaningful change. When combining this information with the smallest worthwhile change (the smallest practical or meaningful change), which was reported at 2% for resting heart rate and 3% for HRV [28], the results can be better contextualized. In particular, in this study, greater changes in HRV than resting heart rate were found in response to a variety of stressors. For certain stressors, able to disrupt resting physiology to a greater extent, the change in both resting heart rate and HRV is well above the smallest worthwhile change (alcohol intake resulted in a 6% change in heart rate and 12% change in HRV, while sickness resulted in a 6% change in heart rate and 10% change in HRV). However, for other stressors, HRV is a more sensitive measure than resting heart rate as only the change in HRV is above the smallest worthwhile change (training resulted in a 1.3% change in heart rate and 4.6% change in HRV while the menstrual cycle resulted in a 1.6% change in heart rate and 3.2% change in HRV). This suggests that HRV is a more practically useful measure than resting heart rate to identify when a change in behavior may be required.

#### 4.2.3. Implications for HRV-Guided Training

HRV-guided training has gained much interest within the research literature [44,45,46]. This concept involves prescribing training and specifically training intensity based on changes in HRV, with generally higher-intensity training being prescribed when HRV is high- and low-intensity training when HRV is low. Such decisions are made based on changes above and below threshold values, constructed at an individual level. Indeed in a recent systematic review and meta-analysis [47], it was shown that, across eight studies, HRV-guided training had a significant, medium-sized positive effect on the improvement of sub-maximal physiological parameters. Furthermore, there were fewer non-responders to HRV-guided training, meaning that the use of this approach induced more consistent favorable effects. However, many studies that investigated HRV-guided training, scheduled enforced rest days rather than scheduling them when required based on individual HRV responses. For example, in [45], the authors enforced one rest day every week, ensuring the same amount of training and rest days for the HRV-guided training group and traditional training group. Given these data and observed sensitivities of both HRV and resting heart rate, the next step with HRV-guided training may be to alter training intensity based on substantial changes in HRV and to prescribe full rest days based on substantial changes in resting heart rate.

### 4.3. Limitations

Despite the large, heterogeneous sample of participants and five-year-long data collection period, there are limitations in our study. In terms of data collection, using a validated measurement and providing instructions to users does not ensure that such instructions are followed. However, potential validity issues were mitigated by developing additional algorithms able to estimate signal quality and by removing measurements detected as being of poor quality. Additionally, only participants who used the app for several months, with an average use of one year per person, were included. It would be unlikely for an individual to misuse the instrument for such a long period of time. Regarding the questionnaires, users might have skipped the questionnaire or not provided verifiable information. Similarly, the questionnaire used is not a standard questionnaire for exercise or to assess other stressors due to the real-life trade-offs present when collecting data for several years daily. The questionnaire had to be simplified to ensure high compliance over time. In the future, it might be beneficial to integrate other objective methods to report stressors. Finally, our analysis is biased towards health-conscious individuals that intentionally downloaded a resting heart rate and HRV tracking app marketed to recreational and professional athletes, and therefore, our findings apply mostly to this group.

## 5. Conclusions

In this work, we have shown how a camera-based, smartphone app could be used to collect longitudinal data in free-living in a large sample of individuals. Using a simple, one minute measurement upon waking, we could confirm the results of previously published studies as well as provide additional insights on the relationship between resting heart rate, HRV, population-level characteristics, and acute stressors. There are important implications to these findings. HRV is currently used in sports settings as well as for health and fitness tracking in the general population. However, targeting improvements in HRV as intervention goals might not be realistic, given the strong heritability coupled with age and low explained variance associated with lifestyle factors such as physical activity levels. On the other hand, HRV is able to capture day-to-day stressors within individuals with high sensitivity and might therefore be better suited for such day-to-day stress assessment and management. HRV might be used to infer changes in, e.g., training, while resting heart rate might be better suited for observing changes in larger stressors, such as sickness. We conclude that resting heart rate and HRV can effectively be used to quantify individual stress responses across a large range of individual characteristics and stressors. Individual awareness of stress responses might facilitate training guidance, behavioral change, and just-in-time interventions in the future.

## Data Availability

The dataset supporting the conclusions of this article is not available due to privacy reasons.
